# Supplementary material for: Sleep and memory complaints in long COVID: an insight into clustered psychological phenotypes
Source: PeerJ. 2024 Jan 30;12:e16669. doi: 10.7717/peerj.16669 (PMC10836207; doi:10.7717/peerj.16669)
Supplement: Supplemental Information 1 [file peerj-12-16669-s001.docx]

**Appendix 1A (English)**

**# Questionnaire for Phase 1 – Acute COVID-19**

**Demographic data**

- Gender
  - Male
  - Female
- Date of birth: __/__/____

**Do you fell any of the following symptoms? (acute phase)**

- myalgia (body/muscle pain)
- hyposmia/anosmia (loss of smell)
- dysgeusia/ageusia (loss of taste)
- fever
- fatigue
- dry cough
- coriza
- dyspnea (difficulty breathing/shortness of breath)
- sore throat
- diarrhea
- headache
- nausea and/or vomiting
- loss of appetite
- abdominal pain
- cough with mucus (expectoration)
- no symptoms
- I don’t want to answer
- other (specify) ____________________

**Was the use of oxygen necessary?**

- Yes
- No

**Was hospitalization necessary?**

- Yes
- No

**Was intubation necessary?**

- Yes
- No

**Was intensive care unit (ICU) required?**

- Yes
- No

**Have you already taken the COVID vaccine?**

- Yes
- No

**Which vaccine? (Coronavac, Oxford-Astrazeneca)**

- Sinovac/Instituto Butantã - CoronaVac
- AstraZeneca/Oxford (Fiocruz) – Covishield
- Pfizer/BioNTech - BNT162
- Janssen (Johnson & Johnson) - Ad26
- Instituto Gamaleya/União Química - Sputnik V
- Did not informed

**When took the 1st. dose?** _____/____/_____

**Already took the 2nd. Vaccine dose?**

- Yes _____/____/_____
- No

**Appendix 2A (English)**

**# Questionnaire for Phase 2 – Chronic Long COVID**

**Was there a reinfection by COVID?**

- Yes
- No

**Body mass index (BMI)**

- Height: ______
- Weight: ______

**Was the** **use of oxygen necessary?**

- Yes
- No

**Was hospitalization necessary?**

- Yes
- No

**Was intubation necessary?**

- Yes
- No

**Was intensive care unit (ICU) required?**

- Yes
- No

**Do you fell any of the following typical COVID-19 symptoms? (Long COVID)**

- myalgia (body/muscle pain)
- hyposmia/anosmia (loss of smell)
- dysgeusia/ageusia (loss of taste)
- fever
- fatigue
- dry cough
- coriza
- dyspnea (difficulty breathing/shortness of breath)
- sore throat
- diarrhea
- headache
- nausea and/or vomiting
- loss of appetite
- abdominal pain
- cough with mucus (expectoration)
- no symptoms
- I don’t want to answer
- other (specify) ____________________

**In relation to your sleep before COVID-19 disease, how satisfied or dissatisfied are you with your current sleep pattern?**

- very satisfied
- satisfied
- indifferent
- dissatisfied
- very unsatisfied

**Do you feel sad or depressed during most time after COVID-19 infection?**

- Yes
- No

**Do you feel sad or depressed during the last 5 months?**

- Yes
- No

**During the last 2 weeks, how frequent did you feel nervous, anxious, or tense?**

- 0– None of these days
- 1– Many days
- 2– More than half of the days
- 3– Almost all days

**During the last 2 weeks, how frequent was you unable to avoid or control your worries?**

- 0– None of these days
- 1– Many days
- 2– More than half of the days
- 3– Almost all days

**Did you feel any 'new symptoms’ after COVID-19, i.e., symptoms that appear after the disease?**

- Drowsiness during the day
- Memory loss
- Difficulties with daily activities
- Motor difficulty
- Difficulty concentrating
- Did not show any symptoms

**Do you have any comorbidities?**

- Cancer (neoplasia)
- Long-term kidney disease (chronic kidney disease - with or without hemodialysis)
- Disease of the lung or bronchus or nose (chronic or allergic bronchitis or pulmonary emphysema or asthma or sinusitis or allergic rhinitis)
- Type 1 or 2 diabetes (high blood sugar)
- Do you have a heart disease? (myocardial infarction or heart valve disease)
- Do you have high blood pressure? (hypertension)
- Do you have weak immune system? (Immune compromised)
- Smoker (currently or in the past)
- Organ or bone marrow transplantation
- No comorbidity
- Another comorbidity: __________________________

**Have you already taken the COVID vaccine?**

- Yes
- No

**Which vaccine? (Coronavac, Oxford-Astrazeneca)**

- Sinovac/Instituto Butantã - CoronaVac
- AstraZeneca/Oxford (Fiocruz) – Covishield
- Pfizer/BioNTech - BNT162
- Janssen (Johnson & Johnson) - Ad26
- Instituto Gamaleya/União Química - Sputnik V
- Did not informed

**When took the 1st. dose?** _____/____/_____

**Already took the 2nd. Vaccine dose?**

- Yes _____/____/_____
- No
