# Supplementary material for: Sleep and memory complaints in long COVID: an insight into clustered psychological phenotypes
Source: PeerJ. 2024 Jan 30;12:e16669. doi: 10.7717/peerj.16669 (PMC10836207; doi:10.7717/peerj.16669)
Supplement: Supplemental Information 2 [file peerj-12-16669-s002.docx]

**Appendix 1A (Portuguese)**

**# Questionário para Fase 1 – Fase aguda de COVID-19 (Acute COVID-19)**

**Dados demográficos**

- Sexo

( ) Masculino

( ) Feminino

- Data de nascimento: __/__/____

**Vou citar alguns sintomas agora e gostaria que o(a) senhor(a) me falasse se apresentou algum dos seguintes sintomas? (fase aguda)**

( ) dor no corpo/musculares

( ) perda de olfato (cheiro)

( ) perda de paladar (gosto)

( ) febre

( ) fadiga

( ) tosse seca

( ) coriza (nariz escorrendo)

( ) dificuldade para respirar/falta de ar

( ) dor de garganta

( ) diarréia

( ) dor de cabeça

( ) náuseas e/ou vômitos

( ) perda de apetite

( ) dor abdominal

( ) tosse com muco

( ) Não apresentou nenhum sintoma

( ) Não quis responder

( ) Tive outro sintoma (especifique)

**Precisou usar oxigênio?**

- Sim
- Não

**Você foi hospitalizado?**

- Sim
- Não

**Você foi intubado?**

- Sim
- Não

**Você precisou internação em unidade de Terapia Intensiva (UTI)?**

- Sim
- Não

**Você já tomou vacina para COVID?**

- Sim
- Não

**Qual vacina? (Coronavac, Oxford-Astrazeneca)**

- Sinovac/Instituto Butantã - CoronaVac
- AstraZeneca/Oxford (Fiocruz) – Covishield
- Pfizer/BioNTech - BNT162
- Janssen (Johnson & Johnson) - Ad26
- Instituto Gamaleya/União Química - Sputnik V
- Não soube informar

**Quando tomou a 1a. dose?** _____/____/_____

**Já tomou a 2a. dose da vacina?**

- Sim _____/____/_____
- Não

**Appendix 2A (Portuguese)**

**# Questionário para Fase 2 – COVID longa (Long COVID)**

**Você teve reinfecção por COVID-19?**

- Sim
- Não

**Precisamos de sua altura e peso, para cálculo do índice de massa corporal (IMC)**

- Altura: ______
- Peso: ______

**Precisou usar oxigênio?**

- Sim
- Não

**Você foi hospitalizado?**

- Sim
- Não

**Você foi intubado?**

- Sim
- Não

**Você precisou internação em unidade de Terapia Intensiva (UTI)?**

- Sim
- Não

**Vou citar alguns sintomas agora e gostaria que o(a) senhor(a) me falasse se apresentou algum dos seguintes sintomas típicos de COVID-19? (Covid longa)**

( ) dor no corpo/musculares

( ) perda de olfato (cheiro)

( ) perda de paladar (gosto)

( ) febre

( ) fadiga

( ) tosse seca

( ) coriza (nariz escorrendo)

( ) dificuldade para respirar/falta de ar

( ) dor de garganta

( ) diarréia

( ) dor de cabeça

( ) náuseas e/ou vômitos

( ) perda de apetite

( ) dor abdominal

( ) tosse com muco

( ) Não apresentou nenhum sintoma

( ) Não quis responder

( ) Tive outro sintoma (especifique)

**Em relação ao seu sono antes de ter a COVID-19, quanto você está satisfeito ou insatisfeito com o seu padrão de sono atual?**

( ) Muito satisfeito

( ) Satisfeito

( ) Indiferente

( ) Insatisfeito

( ) Muito insatisfeito

**Você se sente triste ou deprimido durante a maior parte do tempo após a COVID-19?**

- Sim
- Não

**Você se sente triste ou deprimido nos últimos 5 meses?**

- Sim
- Não

**Durante as últimas 2 semanas, com qual frequência você se sentiu nervoso, ansioso, ou tenso?**

- 0– Nenhum dos dias
- 1– Muitos dias
- 2– Mais da metade dos dias
- 3– Quase todos os dias

**Durante as últimas 2 semanas, com qual frequência você não conseguiu evitar ou controlar as suas preocupações?**

- 0– Nenhum dos dias
- 1– Muitos dias
- 2– Mais da metade dos dias
- 3– Quase todos os dias

**Você sente algum dos sintomas novos após a COVID-19, isto é, sintomas que apareceram após a doença?**

- Sonolência durante o dia
- Perda de memória
- Dificuldade nas atividades diárias
- Dificuldade motora
- Dificuldade de concentração
- Não senti nenhum desses sintomas

**Você tem alguma comorbidade?**

- Câncer (neoplasia)
- Doença renal de longo prazo (doença crônica renal – com ou sem hemodiálise)
- Doença pulmonar, ou nos brônquios ou nariz (bronquite crônica ou alérgica, ou enfisema pulmonar, ou asma, ou sinusite, ou rinite alérgica)
- Diabetes Tipo 1 ou Tipo 2 (açúcar alto no sangue)
- Você tem doença no coração? (infarto do miocárdio, doença valvular cardíaca)
- Você tem pressão alta? (hipertensão)
- Você tem problema no Sistema imune? (imunocomprometido)
- Você é fumante? (atualmente ou no passado)
- Realizou transplante de órgão ou de medula óssea?
- Sem comorbidade
- Outra comorbidade: __________________________

**Você já tomou vacina para COVID?**

- Sim
- Não

**Qual vacina? (Coronavac, Oxford-Astrazeneca)**

- Sinovac/Instituto Butantã - CoronaVac
- AstraZeneca/Oxford (Fiocruz) – Covishield
- Pfizer/BioNTech - BNT162
- Janssen (Johnson & Johnson) - Ad26
- Instituto Gamaleya/União Química - Sputnik V
- Não soube informar

**Quando tomou a 1a. dose?** _____/____/_____

**Já tomou a 2a. dose da vacina?**

- Sim _____/____/_____
- Não
